# Indication of Cognitive Change and Associated Risk Factor after Thoracic Surgery in the Elderly: A Pilot Study

**DOI:** 10.3389/fnagi.2017.00396

**Published:** 2017-12-05

**Authors:** Kay Kulason, Rui Nouchi, Yasushi Hoshikawa, Masafumi Noda, Yoshinori Okada, Ryuta Kawashima

**Affiliations:** ^1^Department of Advanced Brain Science, Institute of Development, Aging and Cancer, Tohoku University, Sendai, Japan; ^2^Creative Interdisciplinary Research Division, Frontier Research Institute for Interdisciplinary Science, Tohoku University, Sendai, Japan; ^3^Human and Social Response Research Division, International Research Institute of Disaster Science, Tohoku University, Sendai, Japan; ^4^Department of Thoracic Surgery, School of Medicine, Fujita Health University, Toyoake, Japan; ^5^Department of Thoracic Surgery, Institute of Development, Aging and Cancer, Tohoku University, Sendai, Japan; ^6^Division of Developmental Cognitive Neuroscience, Institute of Development, Aging and Cancer, Tohoku University, Sendai, Japan

**Keywords:** POCD, mental health, GHQ, prevent, cognitive decline, thoracic

## Abstract

**Background:** This pilot study investigated the effects of partial pulmonary lobectomy lung surgery on cognitive functions of elderly Japanese patients. It is recognized that elderly patients undergoing surgery have increased risk of Postoperative Cognitive Decline (POCD), a condition in which learning, memory, and processing speed is greatly reduced after surgery. Since elderly patients are more likely to exhibit symptoms of POCD, the incidence is increasing as the population receiving surgery is aging.

**Methods:** Cognitive function was measured for all subjects (*n* = 12) before and after surgery using three different cognitive tests: Mini-Mental Status Exam-Japanese (MMSE-J), Frontal Assessment Battery (FAB), and a computerized Cogstate Brief Battery (CBB). Changes in these measures indicate changes in cognitive function. In addition, the 12-item General Health Questionnaire (GHQ-12), the Geriatric Depression Scale (GDS), and the 5-item Quality of Life questionnaire (QOL-5) were administered at each time point to measure mental and emotional state. Changes in outcome measures were analyzed via Wilcoxon signed-rank test. Exploratory correlation analysis was conducted using Spearman’s rho.

**Results:** Data show a decline in detection (DET; *p* = 0.045) and identification (IDN; *p* = 0.038). Spearman’s correlation coefficient show a significant correlation between postoperative DET scores and postoperative IDN scores (ρ = 0.78, *p* = 0.005), a significant correlation between change in IDN and baseline GHQ-12 scores (ρ = -0.595, *p* = 0.027), and a significant correlation between change in one-back (OBK) scores and duration of anesthesia (ρ = -0.72, *p* = 0.012).

**Discussion:** This was the first report to examine cognitive decline after major thoracic surgery in Japanese patients. Previous studies have evidenced that POCD is a common phenomenon after surgery, and that age is a major risk factor. The CCB measured significant change in two cognitive domains: attention and psycomotor function. This study clarified that decline in cognition is detectable in certain measures after thoracic surgery in the elderly Japanese patient population. Additionally, longer anesthetic exposure may negatively impact attention and working memory, and preoperative mental wellbeing is a possible predictor of POCD. These preliminary results have important implications and support the need for future studies.

## Introduction

Symptoms including memory loss and lack of concentration often occur in patients who have undergone surgery (Bedford, 1955). Although not officially diagnosed, these symptoms are part of a condition called postoperative cognitive decline (POCD) (Tsai et al., 2010) and is often described in literature as acute (1 week), intermediate (3 months), and long-term (1+ years) (Leung and Sands, 2009). POCD has been reported in the literature since the 1950s, and recent studies suggest that anesthesia is a potential culprit (Bedford, 1955; Stratmann, 2011). Bedford (1955) published a retrospective review of 1193 elderly patients who, over a 5-year period, underwent surgery with general anesthesia. The review concluded that cognitive problems correlated with anesthetic agents and hypotension, and that ‘operations on elderly people should be confined to unequivocally necessary cases.’ Separately, an international multicenter study on POCD reported memory impairments in 26% of patients 60 years and older. Deficits in cognitive functions were reported to last anywhere from months to years (Moller et al., 1998). While most incidents of POCD naturally recover 6 months after surgery, in nearly 2% of POCD cases symptoms can last until death (Bedford, 1955). POCD is also a problem as it is also strongly associated with premature departure from the labor market (Steinmetz et al., 2009). Furthermore, people with POCD are at higher risk of death within the first year after surgery (Monk and Price, 2011).

In fact, studies assessing general health and quality of life have found that changes in cognitive function are correlated with physical health, emotional health, and quality of life (Launer et al., 1995; Benito-León et al., 2002; Colcombe and Kramer, 2003; Hopkins et al., 2004). A decline in quality of life and general health are associated with declines in cognitive functions and depression (Lindholt et al., 2002; Jones et al., 2006). Interestingly, associations between change in test performance and age, physical disability, and a number of depressive symptoms have been reported (Stockton et al., 2000). Preoperative symptoms of depression have also been associated with the development of postoperative delirium (Leung et al., 2011). Although POCD and delirium are believed to be separate entities, their symptoms are similar and their relationship has yet to be determined (Deiner and Silverstein, 2009; Tsai et al., 2010). Age is nevertheless the biggest risk factor for POCD (Moller et al., 1998; Monk et al., 2008). Over the past 20 years, the number of older people undergoing surgical procedures has increased faster than the population is aging (Etzioni et al., 2003; Sauër et al., 2009). Consequently, maintaining and preventing cognitive decline in older adults after surgery is drawing increasing attention (Schaie et al., 1987; Baltes et al., 1989; Simões, 1998; Ball et al., 2002; Hedden and Gabrieli, 2004; Willis et al., 2006; Bissig and Lustig, 2007; Smith et al., 2009; Tucker-Drob et al., 2009; Zelinski, 2009; Lövdén et al., 2010; Mowszowski et al., 2010; Williams and Kemper, 2010; Martin et al., 2011; Tardif et al., 2011; Fernández-Prado et al., 2012).

Changes in cognitive function in the elderly Japanese population have previously been examined (Saito et al., 2013; Tachibana et al., 2015). Tachibana et al. (2015) focused on the effect of administering desflurane anesthesia vs. sevoflurane anesthesia in all surgeries lasting longer than 4 h. Additionally, Tachibana et al. (2015) measured cognitive function using only the Mini-Mental Status Exam (MMSE) 24h before and after surgery. Saito et al. (2013), on the other hand, examined patients undergoing carotid endarterectomy for ipsilateral cervical internal carotid artery stenosis (≥70%) and analyzed neuropsychiatric data on cognitive function taken preoperatively and 1 month postoperatively with brain proton MR spectroscopy. The main aim of this present study is to examine the cognitive changes after major thoracic surgery and utilizes the MMSE in conjunction with several other measures including a computerized battery to detect changes in cognitive function. Cognitive functions were measured via the conventionally used Mini-Mental Status Exam-Japanese (MMSE-J) and the Frontal Assessment Battery (FAB) in conjunction with the Cogstate Brief Battery (CBB). Additionally, the 12-item General Health Questionnaire (GHQ-12), the Geriatric Depression Scale (GDS), and the 5-item Quality of Life questionnaire (QOL5) were administered at each time point to measure mental and emotional state. Testing for this present study was administered approximately 1 day before surgery and 1 week after surgery.

This study investigated possible risk factors of POCD that could eventually be targeted to reduce the risk of and prevent declines in cognitive functions after surgery. Several studies have investigated POCD risk factors, and they have concluded that age is the largest risk (Salthouse, 1996, 2003; Royall et al., 2004; Coppin et al., 2006; Yakhno et al., 2007). Carrying the APOE4 genotype and inflammation are also believed to be risk factors of POCD. Studies have suggested that the effects of APOE are mediated through alterations in lipid transport in regenerating neurons, proinflammatory cytokine release from activated microglia, amyloid precursor protein metabolism, increased blood brain barrier permeability, alterations in platelet function, and systemic inflammation (Tsuang and Bird, 2002; Parihar and Hemnani, 2004; Moretti et al., 2005). Unfortunately, carrying the APOE4 gene and inflammation are not factors that can be easily treated or prevented. Moreover, anesthesia alone has been shown to increase the level of proinflammatory cytokines (Cao et al., 2012; Wu et al., 2012; Žura et al., 2012). Additionally, depression has been linked with cognitive decline. A review of a decade of the literature concluded that major depression has been associated with impaired cognitive functioning (Hammar and Årdal, 2009). Therefore, it was hypothesized that time under anesthesia and preoperative depressive symptoms would correlate with cognitive decline.

## Materials and Methods

### Participants

A total of 12 volunteers (six males, six females) were recruited from respiratory patients undergoing lung surgery with general anesthesia. Participant demographics are noted in **Table 1**. Respiratory patients who underwent partial pulmonary lobectomy lung surgery to remove tumor growths were recruited from Tohoku University Hospital. A doctor of thoracic surgery from the Tohoku University Hospital referred participants to the study. Participants were native Japanese speakers who self-reported to be right-handed and were 60 years and older (mean age 70.16 ± 6.07 years). Participants were unconcerned with their memory functions.

**Table 1:** Demographic information.

Gender	5 males, 6 females
Mean Age (years)	70.16 ± 6.07
Race/Ethnicity	Japanese
Procedure	Partial pulmonary lobectomy
Location of procedure	Tohoku University Hospital
Mean anesthesia duration (min)	257.45 ± 70.14
**Mean anesthesia administered**	
-Remifentanil (mg)	4.31 ± 2.47
-Fentanyl (mg)	0.28 ± 0.13

Patients admitted into the hospital were informed about the study prior to surgery. Interested patients received both a written and a verbal explanation of the study. Prior to participating in the study, all subjects were requested to sign the informed consent form. The protocol of the study and the consent form were approved by the Ethics Committee of Tohoku University Graduate School of Medicine. This study was registered with the University Hospital Medical Information Network (UMIN) Clinical Trial Registry (UMIN000019832).

### Cognitive Function Measures

A total of three cognitive tests were administered to participants before (1 day) and after surgery (∼1 week, 5–17 days between assessment). The Mini-Mental State Examination-Japanese (MMSE-J) was administered to the participants after receiving consent. The MMSE-J is a 30-point cognitive test used extensively in both the clinical and research settings to measure cognitive impairment (Pangman et al., 2000). Conventionally, clinicians consider a person’s MMSE-J score along with their history, a physical health exam, symptoms, and results from other tests to assess Dementia. A higher MMSE-J score indicates better cognitive performance. The MMSE is the most common mental status test used to determine POCD (Folstein et al., 1975; Tombaugh and McIntyre, 1992; Wang et al., 2014). For test–retest intervals of 2 months or less, the MMSE has been shown to have good reliability (Folstein et al., 1975; Helkala et al., 2002).

Following the MMSE-J, the participants were administered the Frontal Assessment Battery (FAB). The FAB is an 18-point cognitive test that is commonly performed at bedside or in a clinical setting to help measure cognitive impairment in executive functions (Dubois et al., 2000; Kugo et al., 2007; Nakaaki et al., 2007). Again, higher scores indicate better performance. The Japanese translation of the FAB has previously been shown to be comparable to the original English versions and has a good test-retest reliability at 3-weeks (Kugo et al., 2007; Nakaaki et al., 2007). Both the MMSE and FAB are commonly administered in studies determining cognitive decline (Brown et al., 2010; Bugalho et al., 2013; Barulli et al., 2015).

After the FAB, participants were tested using a laptop running CBB to measure the speed of processing, visual attention, visual learning and memory, and attention and working memory. This computerized battery has been shown to effectively determine cognitive decline (Maruff et al., 2009, 2013; Sauër et al., 2009; Brown et al., 2010; Lim et al., 2012) and has also been shown to be effective in Japan (Yoshida et al., 2011). Previous studies on the test-retest reliability of the CBB have indicated that there are no retest-related increases in scores after 1 month. However, the retest scores have been shown to increase at 1-week or less (Maruff et al., 2009, 2013; Lim et al., 2012). The four CBB tasks have previously been described in detail (Collie et al., 2002; Falleti et al., 2006; Maruff et al., 2013) and are summarized here.

A single playing card is presented in the center of the computer monitor for each trial of each of the four tasks. The values, color, and suit of the playing cards were determined by the requirements of each task. For each of the four tasks, participants were required to respond either “Yes” or “No” by pressing the “K” (yes) or “D” (no) key on the keyboard as quickly and accurately as possible. While the “K” and “D” keys were specifically identified to the participants, the keys surrounding “K” (e.g., U, I, O, J, L, M, “,”, “.”) and “D” (e.g., W, E, R, S, F, X, C, V) also recorded responses during the task. At the beginning of each task, task rules were presented on the computer monitor and were also explained verbally to the participant. This was followed by an interactive demonstration in which participants practiced the task. After completing the practice trial, the participant was again reminded of the task question and started the recorded, full-length task. For each of the four tasks, the reaction time and accuracy were recorded and expressed as mean reaction time (in milliseconds) and accuracy (proportion correct). Cogstate has selected a single representative performance measure for each task on the basis that it comes with a normal data distribution, has no floor or ceiling effects, does not have restricted range, and has good reliability, stability, and sensitivity to change (Fredrickson et al., 2010; Hammers et al., 2011). Each of the four tasks from the CBB is described in order below. The four tasks were always presented in the same order.

The Detection (DET) task measures psychomotor function by recording reaction times. The participant was required to attend to the card in the center of the screen and respond to the question: “Has the card turned over?” Participants were instructed to press the “Yes” key as soon as the card turned face up to reveal a generic joker card. The same joker card was presented in each trial. The task ended after 35 correct trials. The primary performance measure for this task was reaction time in milliseconds (speed), which was normalized using a log_10_ transformation. A lower score indicates better performance. The sign was changed when calculating change in score so that a greater negative change indicates worse performance postoperatively.

The Identification (IDN) task is a choice reaction time test measures visual attention. The participant must attend to the card in the center of the screen and respond to the question “Is the card red?” by pressing the “Yes” and “No” buttons. The face of the displayed cards was either red or black jokers in equivalent numbers which appeared in random order. These joker cards were different to that of the joker displayed in the DET task. The task ends after 30 correct trials. Anticipatory responses were excluded, and another trial was given so that all participants completed the 30 trials. The primary performance measure was reaction time in milliseconds (speed), which was normalized using a log_10_ transformation. A lower score indicates better performance. The sign was changed when calculating change in score so that a greater negative change indicates worse performance postoperatively.

The One Card Learning (OCL) task is a continuous visual recognition learning task that assesses visual learning within a pattern separation model (Yassa et al., 2010). Theoretical models of pattern separation model specify that information is organized in orthogonal and distinct non-overlapping representations so that the memories can be stored rapidly without interference (Norman and O’Reilly, 2003). The task requires the participant to attend the card in the center of the screen and respond to the question “have you seen this card before in this task?” using the “Yes” and “No” buttons. Playing cards numbered from 1 and 13, minus face-cards, were displayed. Six cards are drawn at random from the deck and repeated throughout the task. These cards are interspersed with distractor cards (non-repeating cards). The task ends after 80 trials, which do not reschedule for post-anticipatory correct trials. The primary performance measure for this task was the proportion of correct answers (accuracy), which was normalized using an arcsine square-root transformation. A lower score indicates worse performance, therefore a greater negative change in score after surgery indicates worse performance postoperatively.

The One-Back (OBK) task is a task of working memory and attention. The participants must attend to the card in the center of the screen and respond to the question “is the card the same as that on the immediately previous trial?” using the “Yes” and “No” buttons. The same deck of cards from the OCL task is used. The task ends after 30 correct trials. Correct but post-anticipatory responses led to the scheduling of an extra trial. The primary performance measure for this task was the proportion of correct answers (accuracy), which was normalized using an arcsine square-root transformation. A lower score indicates worse performance, therefore a greater negative change in score after surgery indicates worse performance postoperatively.

A series of integrity checks can be applied to the CBB data to ensure that each subject is completing each task properly. For instance, it is expected that the subject performs the quickest on the easiest task (i.e., Detection). It is also expected that accuracy is above chance level for the results to be considered real. For example, if a person performs below 50% accuracy on a task with a choice of 2 responses, they are performing at chance level. As a result, it is impossible to know whether the subject understood the task.

### Psychological Questionnaires

In addition to the above cognitive measures, the GHQ-12, GDS, and QOL-5 were administered immediately after the cognitive assessments before and after surgery to measure mental and emotional state. The GHQ-12 is a self-report measure of psychological morbidity that screens the domains of depression, anxiety, somatic symptoms and social withdrawal. It is routinely used as a unidimensional measure to detect psychiatric disorders. It is widely used in clinical practice (Richardson et al., 2007), epidemiological research (Henkel et al., 2003), and psychological research (Jones et al., 2006). The GHQ-12 consists of six items that are positive descriptions of mood states and six that are negative descriptions of mood states. The questionnaire is structured so that a higher score is an indication that the individual is at higher risk for developing a psychiatric disorder.

The GDS is a self-report measure designed specifically to screen for depression in the elderly population. This short screening measure is geared toward the cognitive and emotional symptoms of depression including feelings of worthlessness, preference for staying at home, and concern about memory problems. The GDS consists of 30 questions in a simple yes/no format. Of the 30 questions, 20 of the questions indicate the presence of depression when answered positively, while the remaining 10 questions indicate the presence of depression when answered negatively (Yesavage et al., 1982–1983).

The QOL-5 is a self-report measure for global and generic quality of life. This 5-item questionnaire has been shown to have internal consistency and sensitivity, and relevant and practical outcome measurement is available for clinical databases (Lindholt et al., 2002). All tests were administered and scored by trained project investigators. The type of general anesthesia administered, the amount of anesthesia administered, the anesthesia duration, the surgery duration, age, and sex were noted.

### Statistical Analysis

This study was designed to determine whether there is a decline in cognition after surgery in elderly lung surgery patients. Before conducting all statistical analysis, we checked gender differences in all tests using Mann–Whitney *U*-test. Change in scores was calculated (post – pre) for each outcome measure. The sign for the change in score of DET and IDN were reversed so that negative numbers reflected poorer performance. In order to compare cognitive function before and after surgery for all participants, we employed a Wilcoxon signed-rank test. This determined whether cognitive functions declined post-surgery. Additionally, Spearman’s correlation coefficients were used to examine correlations between test scores and covariates including questionnaire scores, the amount of anesthesia administered, and duration of surgery. The level of significance was set at *p* < 0.05. One participant was removed from analysis for failing three of the five CBB integrity checks of the two testing sessions. Statistical analysis was conducted using RStudio (version 3.2.4 [2016-03-10]).

## Results

There were no significant differences in all test scores between genders at both baseline and follow-up. Participants scored similarly on all outcome measures at baseline, and significant changes were detected for two CBB cognitive domain measures post-surgery—the IDN and DET scores (**Table 2**). In a separate representation of the data, changes in cognitive test outcome measures for each subject are listed in **Table 3**. One subject was removed from analysis for failing 3 CBB integrity checks over the two sessions, which indicated a lack of understanding of the task, or not completing the task seriously. Differences between pre-surgery and post-surgery scores were analyzed via Wilcoxon signed-rank tests. Data show a decline in DET (*p* = 0.045) and IDN (*p* = 0.038, **Table 2**). While some individuals have a positive change in their DET and IDN scores post-surgery from baseline, the overall trend of the group is a decline in DET and IDN (**Table 3**). No significant changes were apparent in the other test score outcome measures. Data does not indicate that there is a global decline in cognitive functions after thoracic surgery. Additionally, Spearman’s correlation coefficient indicates a significant relationship between postoperative DET scores and postoperative IDN scores (ρ = 0.78, *p* = 0.005, Supplementary Table 2). There are no significant correlation between age and change in DET, nor is there a significant correlation between age and change in IDN (Supplementary Table 1).

**Table 2:** Primary outcome measure scores.

Primary	Pre-surgery	Pre-surgery	Post-surgery	Post-Surgery	Between score
Outcome	Baseline Scores	Baseline Score	Follow-up Scores	Follow-up Score	*p*-value
Measure	Mean (±SD)	Range	Mean (±SD)	Range	
MMSE	27.50 (±1.17)	26–29	27.92 (±1.88)	26–30	0.144
FAB	13.25 (±1.92)	11–16	13.00 (±1.41)	11–15	0.209
DET	86.17 (±12.08)	65–107	93.08 (±7.17)	84–103	0.045^∗^
IDN	96.58 (±6.54)	83–104	99.75 (±3.70)	94–104	0.038^∗^
OCL	99.75 (±3.60)	94–107	99.00 (±5.74)	89–106	0.347
OBK	101.83 (±5.02)	95–107	102.58 (±7.14)	94–116	0.311
GHQ12	3.25 (±2.83)	0–7	3.17 (±3.07)	0–9	0.452
GDS	4.17 (±3.51)	1–8	3.75 (±3.57)	0–6	0.130
QOL5	13.17 (±4.75)	6–19	14.67 (±4.62)	9–21	0.224

*DET and IDN measure reaction time in milliseconds, so higher scores indicate poorer performance. The OCL and OBK measures accuracy, and the MMSE and FAB scoring methods add points for every correct response. Therefore, higher scores indicate better performance. As for the health questionnaire, a higher GHQ12 score indicates poorer overall psychiatric health, while higher scores for GDS and QOL5 indicate better psychiatric health. Wilcoxon signed-rank tests analyzed for differences between Pre-surgery and Post-surgery scores. There was a significant decline in post-surgery DET (*p* = 0.045) and IDN (*p* = 0.038) scores compared to baseline. ^∗^Indicates significant difference between pre-surgery and post-surgery score (*p* < 0.05)*.

**Table 3:** Individual and mean (±SD) change in cognitive test scores (post-pre).

ID	ΔMMSE	ΔFAB	ΔDET	ΔIDN	ΔOCL	ΔOBK
sub1	0	1	-18	-2	-4	0
sub2	2	1	-22	-8	6	2
sub3	3	1	-7	-2	-6	-6
sub4	1	-2	4	0	1	4
sub5	0	-2	-5	1	-7	3
sub6	2	-1	-5	-2	9	9
sub7	0	0	10	4	1	0
sub8	1	1	5	1	5	-9
sub9	-1	-1	-28	-19	-1	0
sub10	0	0	-15	-5	-11	-3
sub12	-4	3	3	-3	-3	0
Mean Δ (±SD)	0.36 (±1.86)	0.09 (±1.51)	-7.09 (±12.30)^∗^	-3.18 (±6.15)^∗^	-0.91 (±6.06)	0.00 (±5.56)

*Change in outcome measures was calculated by subtracting the pre-surgery scores from the post-surgery scores. The sign for change in DET and change in IDN were reversed because a lower score indicates better performance for these primary outcome measures. All negative values in this table indicate poorer performance during the post-surgery session. Subject 11 was removed from the analysis. ^∗^Indicates significant difference between pre-surgery and post-surgery score (p < 0.05)*.

**FIGURE 1:**
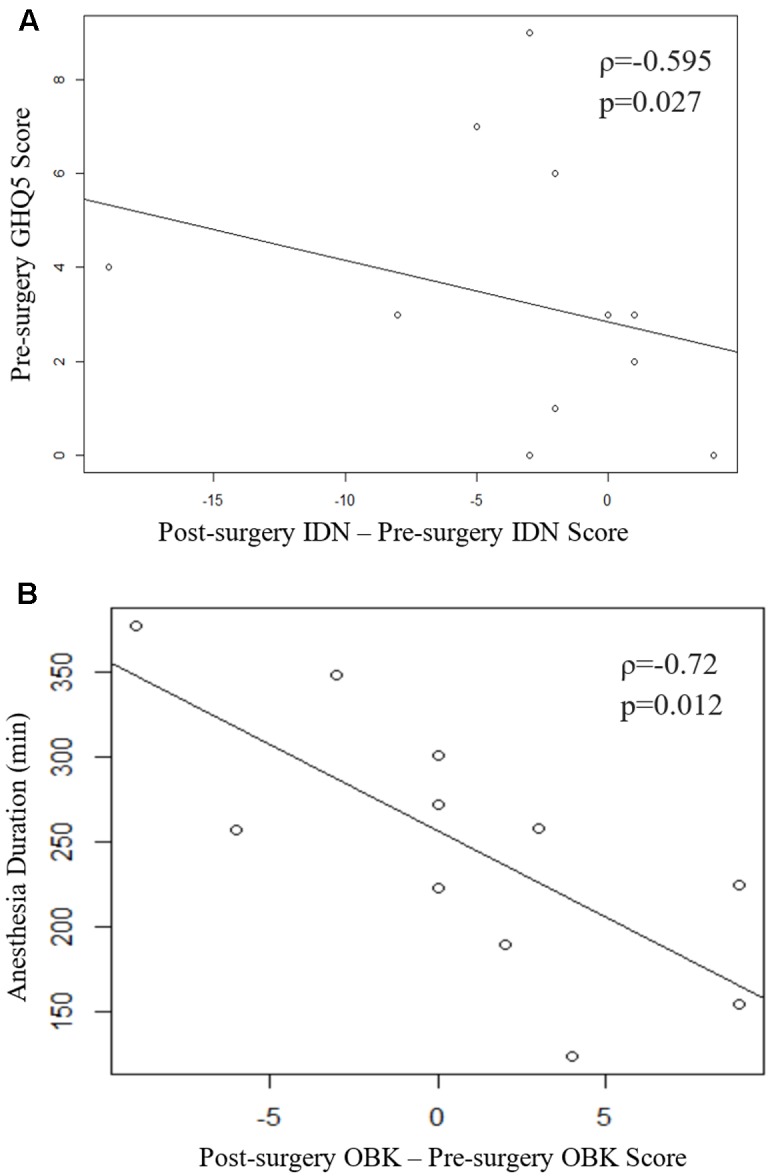
**(A)** Scatter plot of the change in IDN score vs pre-surgery GHQ-12. Spearman’s correlation coefficient indicates a significant relationship (ρ = -0.595, *p* = 0.027). **(B)** Scatter plot of the change in OBK scores vs. duration of anesthesia. Spearman’s correlation coefficient indicates a significant relationship (ρ = -0.72, *p* = 0.012). One subject was removed from analysis for failing 3 of the internal CBB integrity checks over the two sessions.

Spearman’s correlation coefficient also indicates a significant relationship between the change in IDN scores and baseline GHQ-12 scores (ρ = -0.595, *p* = 0.027; **Figure 1A**). Higher GHQ-12 scores were associated with greater IDN score declines after thoracic surgery. No other significant correlations were apparent between change in test scores and baseline questionnaire scores (Supplementary Table 1). Additionally, change in OBK scores were significantly correlated with anesthetic duration (ρ = -0.72, *p* = 0.012; **Figure 1B** and Supplementary Table 1). The longer the duration of anesthesia, the greater the decline in OBK score after surgery. A greater negative change in OBK indicates a decline in attention and working memory cognitive function as measured by the OBK task.

## Discussion

This pilot study was designed to investigate possible, and potentially treatable, risk factors of cognitive decline after major surgery in the elderly Japanese patient population. As previously mentioned, age has been indicated as a major risk factor for POCD and is found in approximately 26% of individuals over 60 years old (Moller et al., 1998). This was the first study to investigate the correlation between depressive symptoms and cognitive decline after surgery. Additionally, it was the first study to examine the effects of thoracic surgery on cognitive functions for the Japanese elderly population.

Preliminary results revealed a decline in both DET and IDN scores after surgery. Previous studies on the test–retest reliability of the CBB have indicated that there are no retest-related increases in score with 1-month intervals. However, at 1-week intervals, the second test scores have been shown to slightly increase (Falleti et al., 2006; Hammers et al., 2011). Despite this fact, our data showed significantly lower DET and IDN scores after surgery compared to baseline. The decline in these scores, especially with the expected increase in scores 1-week post-surgery due to test-retest effects, supports the hypothesis that there is a cognitive decline after thoracic surgery. In fact, this cognitive decline is likely POCD since previous studied regarding POCD have found changes in both attention and processing speed (Tsai et al., 2010)—the same declines indicated by our current data. Nevertheless, a clear conclusion cannot be drawn due to the large SD in our present data for this pilot study, and the small number of subjects. Continued investigations are necessary to determine if POCD is detectable in the elderly thoracic surgery population using the CBB.

There was also a significant correlation between postoperative DET scores and postoperative IDN scores. After surgery, there were significant declines in both DET scores and IDN scores, so there may be a common mechanism that underlie these declines. This may be the case because the task measuring psychomotor function (DET) and visual attention (IDN) share the component of processing speed in that the participant is tasked with responding as quickly as possible in both tasks.

Additionally, Spearman’s correlation coefficient identified a significant correlation between the decline in IDN and baseline GHQ-12 scores. The mental well-being of the patient prior to surgery is potentially a predictor of POCD. Previous studies have found associations between a decline in cognitive function and declines in health and quality of life (Launer et al., 1995; Benito-León et al., 2002; Colcombe and Kramer, 2003; Hopkins et al., 2004). A study conducted by Leung et al. (2005) concluded that preoperative depression was a risk factor for delirium. Delirium is an acute state of disorientation that is characterized by disturbances in attention and awareness. Symptoms include hallucinations and inappropriate communication and/or behavior. In contrast, patients with POCD are oriented, but exhibit declines in one or more neuropsychological domains. Both delirium and POCD feature deficits in attention, so some believe that the phenomena are on the same spectrum and that delirium is a higher grade of POCD (Inouye et al., 1990; Rudolph et al., 2008). Nevertheless, whether they are related events on a continuum, or whether they are distinct events remain unclear. Should the events be related, it would make sense that the results of this study indicate that preoperative GHQ-12 scores are significantly correlated with change in IDN. The finding adds further support to Leung et al.’s (2005) conclusion that preoperative depression is a risk factor for the events on this spectrum. In addition, as noted above, previous findings have indicated that the number of depressive symptoms is associated with changes in test scores and a general decline in cognition (Tsai et al., 2010). The preliminary results of this study indicate a new finding that preoperative mental wellbeing is a predictor of POCD. Therefore, future studies will benefit from gathering more extensive demographic information (i.e., education, marital status, general health).

Interestingly, Spearman’s correlation analysis on the data from the present study suggested a significant relationship between change in OBK scores and anesthetic duration. Longer anesthetic exposure was correlated with a greater negative change in OBK score, which indicates a decline in attention and working memory performance after surgery. It has been generally accepted as a rule that the shorter the duration of the anesthetic agent, the shorter the duration of cognitive impairment in the immediate postoperative period. To date, no definitive evidence has been found for the hypothesis that anesthesia itself causes prolonged POCD. Nor is there sufficient evidence to suggest that anesthetics are neurotoxic (Stratmann, 2011). Nevertheless, the present data adds further support to this belief that anesthesia plays a role in POCD.

In addition, there is no significant effect of gender nor of age in the preliminary data. Contrary to present results, population based data has indicated that performance on neurocognitive tests generally decline with age and tend to decline faster in men (Wiederholt et al., 1993). The likely reason is that the small sample size for this pilot study is not enough to examine gender and age effects.

Together, these results suggest that thoracic surgery leads to a decline in cognitive functions related to visual attention and psychomotor functions. Additionally, extended anesthetic duration may have a negative impact on attention and working memory. The data support our hypothesis that cognitive decline after surgery is correlated with time under anesthesia and preoperative emotional health. Therefore, it may be important to work toward reducing the time spent under anesthesia and to research the unintended effects of different types of anesthesia on different domains of cognitive function. It may also be important to care for the patient’s mental state, in addition to their physical state, to reduce the risk of POCD after surgery.

It is important to consider effects of hypoxia and hypoperfusion on POCD. While hypoxia and hypoperfusion were two of the earliest explanations for postoperative cognitive impairment after surgery, the ISPOCD study examining long-term POCD in the elderly found no statistically significant relationship between hypoxemia or hypotensive episodes and POCD (Moller et al., 1998). Based on the previous study, the hypoxia and hypoperfusion would not affect our results.

This current study does include several limitations. Controlling participant variables (i.e., Excluding those with severe hypertension, Parkinson’s disease, multiple sclerosis, thyroid disease, stroke, heart disease, diabetes, utilizing medications that affect cognitive function, etc.) was not realistic due to the short recruitment period. Also, participants were limited to patients receiving thoracic surgery under general anesthesia, and therefore the results are not applicable to all types of surgery. The study should be repeated in patients undergoing different types of surgery of ranging durations using a variety of anesthetics.

Another limitation to consider is the number of participants. Because the recruitment period was short, the number of participants were severely limited. As previously mentioned, the small number of participants made it impossible to employ the same methods used in previous studies in order to detect POCD. However, this was a pilot study intended to detect whether cognitive decline occurs after thoracic surgery in the Japanese elderly population, and to explore the possible effects of cognitive intervention in the elderly population after surgery. Therefore, the small sample size should be enough to provide the basic groundwork necessary to conduct future investigations. Furthermore, a collection of radiological and biochemical data was not feasible for the present study. These data would add strength to the current findings and deepen understanding. Radiological and biochemical data collection should be considered for future studies. In addition, large sample research using the unification of the anesthesia method during surgery is necessary as a proposal for future research.

The most commonly used composite measure to determine POCD is the MMSE. It is used in 21% of studies, according to a review by (Tsai et al., 2010). The majority of these studies have detected a decline in MMSE scores in subjects determined to have POCD. However, it is likely that the present study detected no change in MMSE scores due to test timing. Studies that find changes in MMSE scores after surgery conduct testing immediately after surgery (1–3 days), rather than after 1 week (Tsai et al., 2010). Again, the POCD studies tend to be conducted in patients undergoing cardiac surgery with an older mean age (Tsai et al., 2010). Nevertheless, the results of this study suggest that it is possible to detect declines in two different domains, processing speed and visual attention, 1 week after surgery.

While cognitive functions can be affected by surgery (Moller et al., 1998), most cognitive functions also decrease with age (Hedden and Gabrieli, 2004). Furthermore, people are living longer, and the elderly population is increasing. Just in Japan, 25% of the population is over 65 years of age. This is the highest proportion in the world and represents a 4% increase between 2011 and 2016 (World Bank, 2017). This increase is also reflected in the surgical population. In fact, over the past 20 years, the number of older people undergoing surgical procedures have increased faster than the population is aging (Etzioni et al., 2003). The results of this study have important implications for the need to conduct further work to investigate cognitive decline post-surgery, and the need to explore treatment methods to ameliorate the effects of cognitive decline after surgery, as well as general cognitive decline, in the elderly population.

## Ethics Statement

Ethical approval was provided by the Institutional Review Board of the Tohoku University Graduate School of Medicine (Ref.2015-1-512). Based on the Declaration of Helsinki, written informed consent was received from each participant.

## Author Contributions

KK, RN, YH, MN, YO, and RK designed developed the study protocol. KK and RN searched the literature, selected cognitive function measures, created manuals to conduct and rate cognitive measures KK and RN wrote the manuscript with YH, MN, YO, and RK. RK also gave advice related to the study protocol. All authors read and approved the final manuscript. KK and RN contributed equally to this work.

## Conflict of Interest Statement

The authors declare that the research was conducted in the absence of any commercial or financial relationships that could be construed as a potential conflict of interest.
