# Supplementary material for: Indication of Cognitive Change and Associated Risk Factor after Thoracic Surgery in the Elderly: A Pilot Study
Source: Front Aging Neurosci. 2017 Dec 5;9:396. doi: 10.3389/fnagi.2017.00396 (PMC5723308; doi:10.3389/fnagi.2017.00396)
Supplement: Supplementary file 1 [file Table_1.PDF]

Supplementary Material

Indication of cognitive change and associated risk factor after thoracic surgery in the elderly: a pilot study.

Authors and Affiliations

Kay Kulason, Rui Nouchi*, Yasuhi Hoshikawa, Masafumi Noda, Yoshinori Okada, Ryuta Kawashima

*correspondence: Corresponding Author: rui.nouchi.a4@tohoku.ac.jp

Supplementary Material

Table 1. The correlation matrix for study outcome measures using change scores.
* Spearman's rank correlation rho is significant at the 0.05 level; ** Spearman's rank correlation rho is significant at the 0.01 level

	Δ MMSE	ΔFAB	ΔDET	ΔIDN	ΔOCL	ΔOBK	Age	Anesthesia Duration (min)	Fentanyl (mg)
ΔMMSE *rho*	1								
p-value									
ΔFAB *rho*	0.34	1							
p-value	0.3								
ΔDET *rho*	0.14	-0.21	1						
p-value	0.68	0.54							

ΔIDN *rho*	0.01	-0.13	0.87**	1					
p-value	0.98	0.7	0.0005						
ΔOCL *rho*	0.49	0.1	0.28	0.05	1				
p-value	0.13	0.8	0.41	0.88					
ΔOBK *rho*	0.14	-0.6	0.08	-0.08	0.37	1			
p-value	0.7	0.052	0.81	0.82	0.26				
Age *rho*	0.38	-0.32	0.05	-0.35	0.55	0.06	1		
p-value	0.25	0.53	0.88	0.3	0.08	0.86			
Anesthesia Duration (min) *rho*	-0.4	0.15	0.15	0.22	-0.28	-0.72	0.25	1	
p-value	0.22	0.67	0.67	0.51	0.41	0.01	0.45		
Fentanyl (mg) *rho*	-0.35	-0.10	-0.55	-0.42	-0.43	-0.31	-0.26	0.19	1
p-value	0.3	0.76	0.08	0.2	0.19	0.35	0.44	0.58	
