# Supplementary material for: Indication of Cognitive Change and Associated Risk Factor after Thoracic Surgery in the Elderly: A Pilot Study
Source: Front Aging Neurosci. 2017 Dec 5;9:396. doi: 10.3389/fnagi.2017.00396 (PMC5723308; doi:10.3389/fnagi.2017.00396)
Supplement: Supplementary file 2 [file Table_2.PDF]

Supplementary Material

Indication of cognitive change and associated risk factor after thoracic surgery in the elderly: a pilot study.

Authors and Affiliations

Kay Kulason, Rui Nouchi*, Yasuhi Hoshikawa, Masafumi Noda, Yoshinori Okada, Ryuta Kawashima

*correspondence: Corresponding Author: rui.nouchi.a4@tohoku.ac.jp

Supplementary Material

Table 2. The correlation matrix for study outcome measures using baseline and follow-up scores.
* Spearman’s rank correlation rho is significant at the 0.05 level; ** Spearman’s rank correlation rho is significant at the 0.01 level

	Baseline GHQ12	Baseline GDS	Baseline QOL5	Baseline MMSE	Baseline FAB	Baseline DET	Baseline IDN	Baseline OCL	Baseline OBK	Follow-up MMSE	Follow-up FAB	Follow-up DET	Follow-up IDN	Follow-up OCL	Follow-up QBK	Age	Anesthetic Duration	Fentanyl
**Baseline**																		
**GHQ12** *rho*	1																	
p-value																		
**GDS** *rho*	0.4	1																
p-value	0.22																	
**Baseline**																		
**QOL5** *rho*	-0.03	0.62	1															
p-value	0.93	0.17																
**Baseline MMSE** rho	-0.54	-0.45	-0.1	1														
p-value	0.09	0.17	0.78															
**Baseline FAB** *rho*	-0.25	0.08	-0.16	0.19	1													
p-value	0.46	0.81	0.63	0.53														
**Baseline DET** *rho*	-0.24	-0.06	0.03	0.15	0.14	1												
p-value	0.49	0.87	0.93	0.64	0.67													
**Baseline IDN** *rho*	-0.1	0.06	0.16	0.28	0.3	0.88**	1											
p-value	0.78	0.86	0.63	0.38	0.35	0.0002												
**Baseline OCL** *rho*	-0.3	0.13	-0.07	0.23	0.1	-0.14	-0.22	1										
p-value	0.37	0.71	0.83	0.47	0.76	0.66	0.49											
**Baseline OBK** rho	-0.38	0.25	0.34	0	0.29	0.11	0.2	0.13	1									
p-value	0.25	0.45	0.3	1	0.36	0.73	0.54	0.68										
**Follow-**	-0.17	-0.16	0.61	0.15	0.12	0.65	0.58	-0.30	0.03	1								

**up MMSE** *rho*																		
p-value	0.63	0.64	0.05	0.65	0.75	0.06	0.06	0.37	0.93									
**Follow-up FAB** *rho*	-0.28	-0.02	0.39	-0.09	0.67	0.07	0.11	-0.10	0.58	0.29	1							
p-value	0.41	0.95	0.23	0.78	0.03	0.83	0.75	0.75	0.06	0.39								
**Follow-up DET** *rho*	0.1	0.08	0.38	-0.25	-0.002	0.28	0.40	-0.57	0.02	0.61	0.14	1						
p-value	0.76	0.82	0.25	0.46	0.99	0.41	0.22	0.07	0.94	0.07	0.69							
**Follow-up IDN** *rho*	0.34	0.38	0.23	-0.28	0.18	0.49	0.61	-0.27	0.03	0.55	0.11	0.78**	1					
p-value	0.31	0.26	0.49	0.41	0.60	0.12	0.05	0.42	0.93	0.08	0.74	0.005						
**Follow-up OCL** *rho*	0.19	0.51	0.07	-0.35	-0.25	0.27	0.10	0.18	0.19	-0.13	-0.18	0.07	0.39	1				
p-value	0.58	0.08	0.83	0.30	0.45	0.41	0.77	0.61	0.58	0.70	0.60	0.83	0.23					
**Follow-up OBK** *rho*	-0.18	0.08	0.26	-0.22	0.40	0.30	0.28	-0.58	0.34	0.38	0.61	0.58	0.41	0.12	1			
p-value	0.6	0.82	0.45	0.51	0.22	0.38	0.40	0.06	0.31	0.25	0.07	0.06	0.21	0.72				
**Follow-up Age** *rho*	0.06	-0.03	-0.79**	-0.25	-0.13	-0.1	-0.23	-0.12	-0.24	-0.54	-0.49	-0.19	-0.29	-0.07	-0.24	1		
p-value	0.86	0.94	0.004	0.46	0.7	0.77	0.5	0.74	0.5	0.09	0.12	0.57	0.40	0.84	0.49			
**Anesthetic**	0.25	0.02	0.4	-0.09	-0.32	0.05	-0.16	0.32	0.005	-0.02	0.11	-0.47	-0.22	0.21	-0.27	-0.53	1	

**Duration** *rho*																		
p-value	0.45	0.96	0.23	0.78	0.34	0.91	0.63	0.34	0.99	1	0.76	0.14	0.52	0.54	0.42	0.09		
**Fentanyl** *rho*	0.59	-0.11	0.09	-0.33	-0.01	-0.33	-0.26	-0.10	-0.26	-0.02	0.10	0.30	0.39	-0.16	-0.03	-0.26	0.19	1
p-value	0.053	0.75	0.80	0.33	0.98	0.31	0.44	0.78	0.44	0.94	0.78	0.37	0.24	0.64	0.93	0.44	0.58	
